# Association between wealth status and health insurance enrollment among women aged 15–49 years in Tanzania: a secondary analysis of the 2022 Demographic and Health Survey

**DOI:** 10.3389/frhs.2026.1896869

**Published:** 2026-08-24

**Authors:** Alphoncina Kagaigai, Malale Tungu, Novatus Julius Bihemo, Jovinary Adam, Amani Anaeli, Pankras Luoga

**Affiliations:** 1 Department of Development Studies, School of Public Health and Social Sciences, Muhimbili University of Health and Allied Sciences, Dar es Salaam, Tanzania; 2 Magu District Council, Mwanza, Tanzania

**Keywords:** health insurance, Tanzania, TDHS, wealth, women

## Abstract

**Background:**

A relationship between wealth status and health insurance enrollment among women in Tanzania is not well established. This study aimed to fill that gap by determining the association between wealth status and health insurance enrollment among women aged 15–49 years in Tanzania.

**Methods:**

The study used secondary data from the 2022 Tanzania Demographic and Health Survey (TDHS). The binary dependent variable was health insurance enrollment. Independent variables included women's sociodemographic characteristics. Descriptive and inferential analyses were conducted. The level of association was measured using Adjusted Odds Ratio (AOR) at a 95% confidence interval (CI). A threshold of *p* < 0.05 was used to determine statistical significance.

**Results:**

Overall, 5.8% (95% CI: 5.1%–6.6%) of women aged 15–49 years in Tanzania were enrolled in health insurance. Logistic regression results indicated that women who belonged to rich households (AOR=2.59; 95% CI: 1.91, 3.53), women aged 35–49 years (AOR=3.86; 95% CI: 2.90, 5.13), women with secondary or higher education (AOR=6.47; 95% CI: 4.17, 10.05), women residing in the Central zone (AOR=1.60; 95% CI: 1.12, 2.28), and women who visited a health facility in the past 12 months (AOR=1.37; 95% CI: 1.14, 1.63) had significantly higher odds of being enrolled. Women who perceived distance to a health facility as not a big problem (AOR=1.70; 95% CI: 1.27, 2.28) and women from female-headed households (AOR=1.29; 95% CI: 1.03, 1.62) also had higher odds of enrollment. Conversely, women who were divorced or separated (AOR=0.50; 95% CI: 0.35, 0.72), women residing in rural areas (AOR=0.68; 95% CI: 0.54, 0.86), and women in the Coastal zone (AOR=0.61; 95% CI: 0.44, 0.86) had significantly lower odds of enrollment.

**Conclusion:**

Health insurance enrollment among women of reproductive age in Tanzania is low. There was a positive association between wealth status and health insurance enrollment. The government and other stakeholders should design targeted interventions, including linking households to social protection programs such as the Tanzania Social Action Fund (TASAF), to improve household wealth and thereby increase health insurance enrollment as the country works toward achieving universal health coverage.

## Background

Globally, Universal Health Coverage (UHC) stands as a central global health priority and a critical objective for nations across all developmental levels (1). Countries are actively working to achieve UHC through various strategies and health financing models, aiming to ensure that every person can access essential health services without enduring financial hardship. This commitment has been reinforced by the World Health Organization (WHO), which through a World Health Assembly resolution has called upon countries to develop robust health financing options to protect populations from catastrophic out-of-pocket (OOP) health expenditures.

The importance of UHC was further reinforced on the international stage in 2015 when the United Nations explicitly included it in the Sustainable Development Goals (SDG 3, Target 3.8). This inclusion set a definitive global target, calling on all countries, particularly Low- and Middle-Income Countries (LMICs), to achieve UHC by the year 2030. Achieving this target hinges on implementing effective financial protection mechanisms that improve access to care and ensure equitable service provision. Health insurance is globally recognised as one of the most reliable and viable financing options for realising the UHC goal, as it provides financial protection and significantly improves access to necessary healthcare services (2). While coverage is generally higher in high-income countries, LMICs continue to struggle with limited enrollment (1, 3). Furthermore, financial vulnerability from medical spending is a gender-sensitive issue, as women experience greater susceptibility to financial distress from healthcare costs, making equitable insurance coverage a key consideration in global health policy.

Despite the strong regional commitment to UHC, health financing efforts in Sub-Saharan Africa (SSA) are severely hindered by alarmingly low health insurance coverage (4, 5). This limited enrollment forces healthcare systems to rely heavily on OOP payments, which consequently serve as a significant financial barrier to accessing healthcare services (5). The continuation of high OOP expenditure undermines the UHC principle of financial protection, pushing many households, especially those in poverty toward financial catastrophe when medical care is needed (5).

The disparities in coverage are particularly stark among women. Studies indicate that only approximately 8.5% of women of reproductive age (WRA) in SSA have access to health insurance (6). While overall coverage for married women between 2010 and 2020 averaged 21.3%, this figure masks massive regional and country-specific variations, with rates ranging from as low as 0.5% to as high as 66.7% across different nations (4). The situation is even more challenging in East Africa, where coverage among WRA is estimated at approximately 7.6%, limiting their access to healthcare services (7, 8). These figures reflect persistent inequalities and a clear failure to reach one of the most vulnerable demographic groups with effective health financing mechanisms (4).

This context of low coverage and financial barriers contributes to sub-optimal healthcare utilisation in SSA. While existing research has identified several factors associated with service use, including socioeconomic status, education, distance to facilities, and trust in the system. Most evidence is based on the general population and often covers limited geographic areas (9). This creates a critical knowledge gap in understanding the specific association between health insurance, socioeconomic status (including wealth), and utilisation among vulnerable groups such as women of reproductive age, highlighting the need for more targeted studies in the region.

Tanzania is actively pursuing UHC and has demonstrated strong political commitment through significant reforms, including the promotion of schemes like the National Health Insurance Fund (NHIF) and the Community Health Fund (CHF) (10). The country has reported both achievements and challenges towards achieving UHC, including developing sustainable financing mechanisms (11). Most recently, the government introduced a mandatory health insurance act aimed at expanding coverage across the population (12). Despite these efforts, health insurance enrollment among women of reproductive age (15–49 years) remains critically low, with analysis of the 2022 Tanzania Demographic and Health Survey (TDHS) data conducted in the present study revealed that only 5.8% (95% CI: 5.1%–6.6%) of women in this age group were enrolled in any health insurance scheme.

Consequently, financial barriers remain the most frequently reported obstacle to accessing healthcare among women of reproductive age in Tanzania. This absence of coverage leads to severe financial hardship, with reports of women experiencing delayed care, denial of services, or prolonged detention in health facilities due to their inability to pay medical bills (13). While health insurance is widely promoted as a viable financing option to improve service accessibility, existing empirical evidence is limited. Previous studies on the association between insurance status, socioeconomic factors (such as wealth), and utilisation among Tanzanian women are often localised, use small sample sizes, and fail to provide nationally representative findings. This study aimed to determine the association between wealth status and health insurance enrollment among women aged 15–49 years in Tanzania. The findings are crucial for strategising the attainment of universal health coverage for Tanzania's population.

## Methods

### Study design

In this study, we analysed secondary data drawn from the 2022 TDHS. The data were collected using a cross-sectional design. The survey is conducted every five years. A detailed description of the methodology and questionnaires used in the survey is available in the final report of the TDHS 2022 (14).

### Study population

The study population comprised a weighted sample of 15,254 women aged 15–49 years who were interviewed during the survey. This included both permanent residents and visitors who had spent a night in the household before the data collection day, consistent with the standard DHS de facto household membership definition. The analysed information was drawn from the women's individual records (IR) file.

### Dependent variable

The dependent variable was health insurance coverage (variable code: v481). Each woman was asked whether she was covered by any health insurance scheme, with possible responses of no or yes, yielding a binary dependent variable. We coded “no” as 0 and “yes” as 1. All women responded to this question and there were no missing values for the dependent variable.

### Independent variables

The primary independent variable was household wealth status derived from the DHS wealth index (v190). The original DHS wealth index comprises five quintiles (poorest, poorer, middle, richer and richest). For this study, the quintiles were collapsed into three categories to facilitate interpretation and ensure adequate observations within each category: poorest and poorer were combined as “poor”, middle remained as “middle”, and richer and richest were combined as “rich”. Other independent variables included age (15–24, 25–34, and 35–49 years), educational level (no formal education, primary education, secondary or higher education), marital status (never married, married, divorced/separated), place of residence (urban/rural), geographical zone (Lake zone, Northern zone, Central zone, Southern zone, Coastal zone), working status (no/yes), distance to the health facility (a big problem/not a big problem), permission to access healthcare (a big problem/not a big problem), self-reported health status (good, moderate to bad), sex of household head (male/female), age of husband/partner (15–24, 25–34, 35–49, 50 years and above), and education of husband/partner (no formal education, primary education, secondary or higher education). Variables were selected based on the existing literature related to the topic (4, 8, 15, 16).

### Data analysis

All data were weighted using the women's individual weight (v005/1,000,000) to account for the complex sampling design and non-response during data collection. Inferential analyses were conducted using the “svyset” command in Stata, specifying the primary sampling unit (cluster variable v021) and sampling strata (v022) alongside the sampling weight, to account for stratification and clustering effects, following analysis guidance from the DHS programme (17). All analyses were conducted using Stata version 18. Multicollinearity among explanatory variables was assessed using Variance Inflation Factors (VIFs). Variables with VIF values below 5 were considered to have no problematic multicollinearity. All variables retained in the final multivariable model had acceptable VIF values. Based on this threshold, the age and educational level of the husband/partner were found to be collinear with marital status. Based on the literature and theoretical relevance, marital status was retained and the husband's age and educational level were excluded from the multivariable model. Multivariable logistic regression analysis was conducted to determine the magnitude of association between independent variables and the dependent variable. A *p*-value threshold of <0.05 was used to determine statistical significance. *Model goodness-of-fit for the multivariable logistic regression was assessed using the Archer-Lemeshow F-adjusted test for survey data.*

## Results

### Sociodemographic characteristics of respondents

This study included a total weighted sample of 15,254 women. Approximately 44.5% belonged to the rich wealth category. The mean age was 29 years (SD = 10), and the majority were aged 15–24 years. Most respondents (53.3%) had primary education. About 60.7% were married, and 64.3% resided in rural areas. More than half (59.5%) were currently working. Approximately 71.2% indicated that distance to a health facility was not a big problem, and 53.0% had visited a health facility in the past 12 months. About 72.0% reported good health status. Further details are shown in Table 1.

**Table 1:** Sociodemographic characteristics of respondents (*N* = 15,254 weighted sample).

Variable	Frequency (*N*)	Percent (%)
Wealth index		
Poor	5,495	36.0
Middle	2,973	19.5
Rich	6,786	44.5
Age groups		
15–24	5,810	38.1
25–34	4,609	30.2
35–49	4,835	31.7
Mean age 29 (SD = 10)		
Educational level		
No formal education	2,450	16.1
Primary education	8,123	53.3
Secondary and beyond	4,681	30.7
Marital status		
Never married	4,047	26.5
Married	9,252	60.7
Divorced/separated	1,955	12.8
Place of residence		
Urban	5,446	35.7
Rural	9,808	64.3
Geographical zones		
Lake zone	5,722	37.5
Northern zone	1,733	11.4
Central zone	1,573	10.3
Southern zone	3,051	20.0
Coastal zone	3,174	20.8
Respondent currently working		
No	6,182	40.5
Yes	9,072	59.5
Distance to health facility: a big problem?		
A big problem	4,393	28.8
Not a big problem	10,861	71.2
Permission to access healthcare: a big problem?		
A big problem	1,099	7.2
Not a big problem	14,155	92.8
Age of husband/partner (*N* = 9,252)		
15–24	597	6.5
25–34	3,024	32.7
35–49	4,058	43.9
50+	1,572	17.0
Husband education level (*N* = 9,202)		
No formal education	1,151	12.5
Primary education	5,785	62.9
Secondary and beyond	2,265	24.6
Sex of household head		
Male	10,918	71.6
Female	4,336	28.4
Number of under-five children		
No child	4,851	31.8
1–2	8,957	58.7
3–4	1,223	8.0
5–15	223	1.5
Household size		
1–4	5,580	36.6
5–6	4,523	29.7
7+	5,151	33.8
Exposure to mass media		
Not at all	4,690	30.7
Less than once a week	3,602	23.6
At least once a week	6,961	45.6
Self-reported health status		
Good	10,980	72.0
Moderate and bad	4,274	28.0
Visited health facility in the past 12 months		
No	7,167	47.0
Yes	8,087	53.0
Covered by health insurance		
No	14,366	94.2
Yes	888	5.8

### Prevalence of health insurance enrollment by women's background characteristics

Table 2 presents cross-tabulations of health insurance enrollment among 15,254 women of reproductive age (15–49 years) in Tanzania, highlighting significant disparities across various sociodemographic factors. Almost all variables, with the exception of self-reported health status, were statistically significantly associated with health insurance enrollment. Wealth index and education were among the strongest predictors. Only 2.1% of women from poor households were enrolled, compared with 10.2% from rich households (*p* < 0.001). Women with secondary or higher education had an enrollment rate of 13.0%, compared with 1.6% for those with no formal education (*p* < 0.001).

**Table 2:** Cross-tabulations of health insurance enrollment among women aged 15–49 in Tanzania.

Variable	Enrolled (%)	95% CI	*P*-value
Wealth index			<0.001
Poor	2.1	[1.6, 2.7]	
Middle	2.6	[2.0, 3.4]	
Rich	10.2	[9.0, 11.7]	
Age groups			<0.001
15–24	4.5	[3.7, 5.5]	
25–34	5.9	[5.0, 7.0]	
35–49	7.3	[6.2, 8.5]	
Educational level			<0.001
No formal education	1.6	[1.0, 2.4]	
Primary education	2.9	[2.5, 3.5]	
Secondary and beyond	13.0	[11.5, 14.7]	
Marital status			<0.001
Never married	7.3	[6.1, 8.7]	
Married	5.6	[4.9, 6.4]	
Divorced/separated	3.9	[2.9, 5.1]	
Place of residence			<0.001
Urban	8.7	[7.4, 10.3]	
Rural	4.2	[3.5, 5.0]	
Geographical zones			0.003
Lake zone	4.3	[3.4, 5.5]	
Northern zone	8.5	[6.1, 11.7]	
Central zone	8.0	[5.4, 11.6]	
Southern zone	5.3	[4.4, 6.4]	
Coastal zone	6.4	[5.1, 8.1]	
Respondent currently working			0.022
No	5.1	[4.3, 6.1]	
Yes	6.3	[5.5, 7.2]	
Distance to health facility: a big problem?			<0.001
A big problem	2.4	[1.9, 3.2]	
Not a big problem	7.2	[6.4, 8.1]	
Permission to access healthcare: a big problem?			<0.001
A big problem	3.8	[2.6, 5.7]	
Not a big problem	6.0	[5.3, 6.7]	
Age of husband/partner			<0.001
15–24	1.0	[0.4, 2.9]	
25–34	4.3	[3.3, 5.7]	
35–49	6.4	[5.3, 7.6]	
50+	7.7	[6.3, 9.5]	
Husband education level			<0.001
No formal education	1.5	[0.8, 2.7]	
Primary education	2.6	[2.1, 3.2]	
Secondary and beyond	15.4	[13.2, 17.8]	
Sex of household head			0.016
Male	5.5	[4.8, 6.2]	
Female	6.7	[5.6, 8.0]	
Number of under-five children			<0.001
No child	7.5	[6.5, 8.8]	
1–2	5.4	[4.6, 6.3]	
3–4	3.1	[2.0, 4.8]	
5–15	0.6	[0.1, 2.9]	
Household size			<0.001
1–4	6.3	[5.3, 7.4]	
5–6	6.9	[5.9, 8.1]	
7+	4.4	[3.6, 5.3]	
Exposure to mass media			<0.001
Not at all	2.5	[1.9, 3.2]	
Less than once a week	4.0	[3.2, 5.0]	
At least once a week	9.0	[7.9, 10.2]	
Self-reported health status			0.198
Good	6.0	[5.3, 6.8]	
Moderate and bad	5.3	[4.5, 6.4]	
Visited health facility in the past 12 months			<0.001
No	4.6	[3.9, 5.4]	
Yes	6.9	[6.0, 7.9]	

**Table 3:** Variance inflation factor results .

Variable	VIF	1/VIF
Age group	2.18	0.459192
Age of husband	2.06	0.485552
Wealth index for urban/rural	1.61	0.622192
Education level	1.59	0.629392
Husband's education level	1.55	0.646764
Mass media exposure	1.43	0.699737
Place of residence	1.37	0.729264
Under-five Children	1.36	0.735282
Household size	1.33	0.752083
Distance to health facility	1.17	0.854949
Geographical zones	1.15	0.866356
Self-medical help	1.08	0.926605
Visited health facility in the last 12 months	1.08	0.929239
Marital status	1.06	0.944767
Currently working	1.05	0.949632
Self reported health status	1.04	0.964228
Sex of household head	1.01	0.992815
Mean VIF	1.36	

Insurance enrollment was also significantly associated with older age (35–49 years), marital status, place of residence, and geographical zone. Women who did not consider distance to a health facility to be a “big problem” had higher enrollment (7.2%) than those who did (2.4%). As the number of under-five children in the household increased, enrollment dropped from 7.5% among those with no children to 0.6% among those with five or more. Women with at least weekly mass media exposure had higher enrollment (9.0%) than those with no exposure (2.5%). Women who had visited a health facility in the previous year also showed higher enrollment (6.9%) compared with those who had not (4.6%). It is noteworthy that the direction of this association may be bidirectional, as health insurance may also facilitate healthcare utilisation.

### Multicollinearity test

To ensure the reliability and stability of the regression estimates, multicollinearity among the explanatory variables was assessed using the Variance Inflation Factor (VIF). Results indicated no evidence of problematic multicollinearity among the independent variables. The VIF values ranged from 1.01 to 2.18, with a mean VIF of 1.36. All VIF values were well below the commonly accepted threshold of 5 (or 10), suggesting that multicollinearity was not a concern and that the regression coefficients were estimated reliably.

### Association between wealth status and health insurance enrollment among women in Tanzania

After controlling for other factors using multivariable logistic regression, women who belonged to rich households had 2.59 times higher odds of being enrolled in health insurance than those from poor households (AOR=2.59; 95% CI: 1.91, 3.53). Women aged 35–49 years had 3.86 times higher odds of enrollment than those aged 15–24 years (AOR=3.86; 95% CI: 2.90, 5.13). Women with secondary or higher education had 6.47 times higher odds of enrollment than those with no formal education (AOR=6.47; 95% CI: 4.17, 10.05).

Women residing in the Central zone had 1.60 times higher odds of enrollment compared with those in the Lake zone (AOR=1.60; 95% CI: 1.12, 2.28). Women who had visited a health facility in the past 12 months had 1.37 times higher odds of enrollment (AOR=1.37; 95% CI: 1.14, 1.63). Women who perceived distance to a health facility as not a big problem had 1.70 times higher odds of enrollment (AOR=1.70; 95% CI: 1.27, 2.28). Women from female-headed households had 1.29 times higher odds of enrollment than those from male-headed households (AOR=1.29; 95% CI: 1.03, 1.62).

Conversely, women who were divorced or separated had 50% lower odds of enrollment than those who were never married (AOR=0.50; 95% CI: 0.35, 0.72). Women residing in rural areas had 32% lower odds of enrollment than urban women (AOR=0.68; 95% CI: 0.54, 0.86). Women in the Coastal zone had 39% lower odds of enrollment compared with those in the Lake zone (AOR=0.61; 95% CI: 0.44, 0.86).

Exposure to mass media, current working status, permission to access healthcare, number of under-five children, household size, and self-reported health status were not significantly associated with health insurance enrollment in the multivariable model. These variables may have attenuated after adjustment due to confounding by wealth status and educational level. Full model results are presented in Table 4.

**Table 4:** Univariable and multivariable logistic regression analysis of health insurance enrollment among women aged 15–49 years in Tanzania.

Variable	COR (95% CI)	*P*-value	AOR (95% CI)	*P*-value
Wealth index				
Poor (ref)	1		1	
Middle	1.22 [0.84, 1.79]	0.301	0.91 [0.61, 1.36]	0.641
Rich	5.29 [3.96, 7.06]	<0.001	2.59 [1.91, 3.53]	<0.001***
Age groups				
15–24 (ref)	1		1	
25–34	1.33 [1.06, 1.67]	0.015	1.67 [1.30, 2.15]	<0.001***
35–49	1.65 [1.29, 2.13]	<0.001	3.86 [2.90, 5.13]	<0.001***
Educational level				
No formal education (ref)	1		1	
Primary education	1.89 [1.24, 2.90]	0.003	1.27 [0.84, 1.93]	0.261
Secondary and beyond	9.39 [6.09, 14.48]	<0.001	6.47 [4.17, 10.05]	<0.001***
Marital status				
Never married (ref)	1		1	
Married	0.75 [0.61, 0.92]	0.007	0.91 [0.71, 1.17]	0.461
Divorced/separated	0.51 [0.37, 0.71]	<0.001	0.50 [0.35, 0.72]	<0.001***
Place of residence				
Urban (ref)	1		1	
Rural	0.46 [0.36, 0.59]	<0.001	0.68 [0.54, 0.86]	0.001***
Geographical zones				
Lake zone (ref)	1		1	
Northern zone	2.05 [1.33, 3.17]	0.001	1.22 [0.80, 1.88]	0.346
Central zone	1.91 [1.17, 3.11]	0.009	1.60 [1.12, 2.28]	0.009**
Southern zone	1.23 [0.89, 1.69]	0.202	0.96 [0.71, 1.30]	0.795
Coastal zone	1.52 [1.07, 2.16]	0.021	0.61 [0.44, 0.86]	0.005**
Respondent currently working				
No (ref)	1		1	
Yes	1.24 [1.03, 1.50]	0.022	0.96 [0.80, 1.16]	0.702
Distance to health facility				
A big problem (ref)	1		1	
Not a big problem	3.09 [2.34, 4.08]	<0.001	1.70 [1.27, 2.28]	<0.001***
Permission to access healthcare				
A big problem (ref)	1		1	
Not a big problem	1.59 [1.06, 2.39]	0.026	1.07 [0.72, 1.58]	0.744
Sex of household head				
Male (ref)	1		1	
Female	1.25 [1.04, 1.50]	0.016	1.29 [1.03, 1.62]	0.025**
Number of under-five children				
No child (ref)	1		1	
1–2	0.70 [0.58, 0.85]	<0.001	0.91 [0.73, 1.14]	0.379
3–4	0.40 [0.25, 0.64]	<0.001	0.82 [0.52, 1.35]	0.436
5–15	0.07 [0.02, 0.37]	0.002	0.39 [0.06, 2.31]	0.300
Household size				
1–4 (ref)	1		1	
5–6	1.11 [0.89, 1.38]	0.352	1.14 [0.90, 1.44]	0.265
7+	0.68 [0.53, 0.88]	0.003	0.91 [0.68, 1.23]	0.548
Exposure to mass media				
Not at all (ref)	1		1	
Less than once a week	1.61 [1.16, 2.25]	0.005	0.91 [0.66, 1.26]	0.575
At least once a week	3.83 [2.89, 5.08]	<0.001	1.27 [0.96, 1.69]	0.090
Self-reported health status				
Good (ref)	1		1	
Moderate and bad	0.88 [0.73, 1.07]	0.198	1.05 [0.87, 1.27]	0.600
Visited health facility last 12 months				
No (ref)	1		1	
Yes	1.54 [1.29, 1.84]	<0.001	1.37 [1.14, 1.63]	0.001***

AOR, adjusted odds ratio; CI, confidence interval; COR, crude odds ratio.

A single, consistent threshold of *p* < 0.05 was used to determine statistical significance throughout.

** *p* < 0.01.

*** *p* < 0.001.

The final multivariable model demonstrated an adequate goodness-of-fit (Archer-Lemeshow test, *p* > 0.05). However, the Pseudo R2 was not perfomed on this model since the dataset has been declared for survey observation.

### The Archer-Lemeshow F-adjusted test

Number of observations = 15,254
F-adjusted test statistic F (9,559) = 2.062
Prob > F = 0.031

### Forest plot

**Figure 1:**
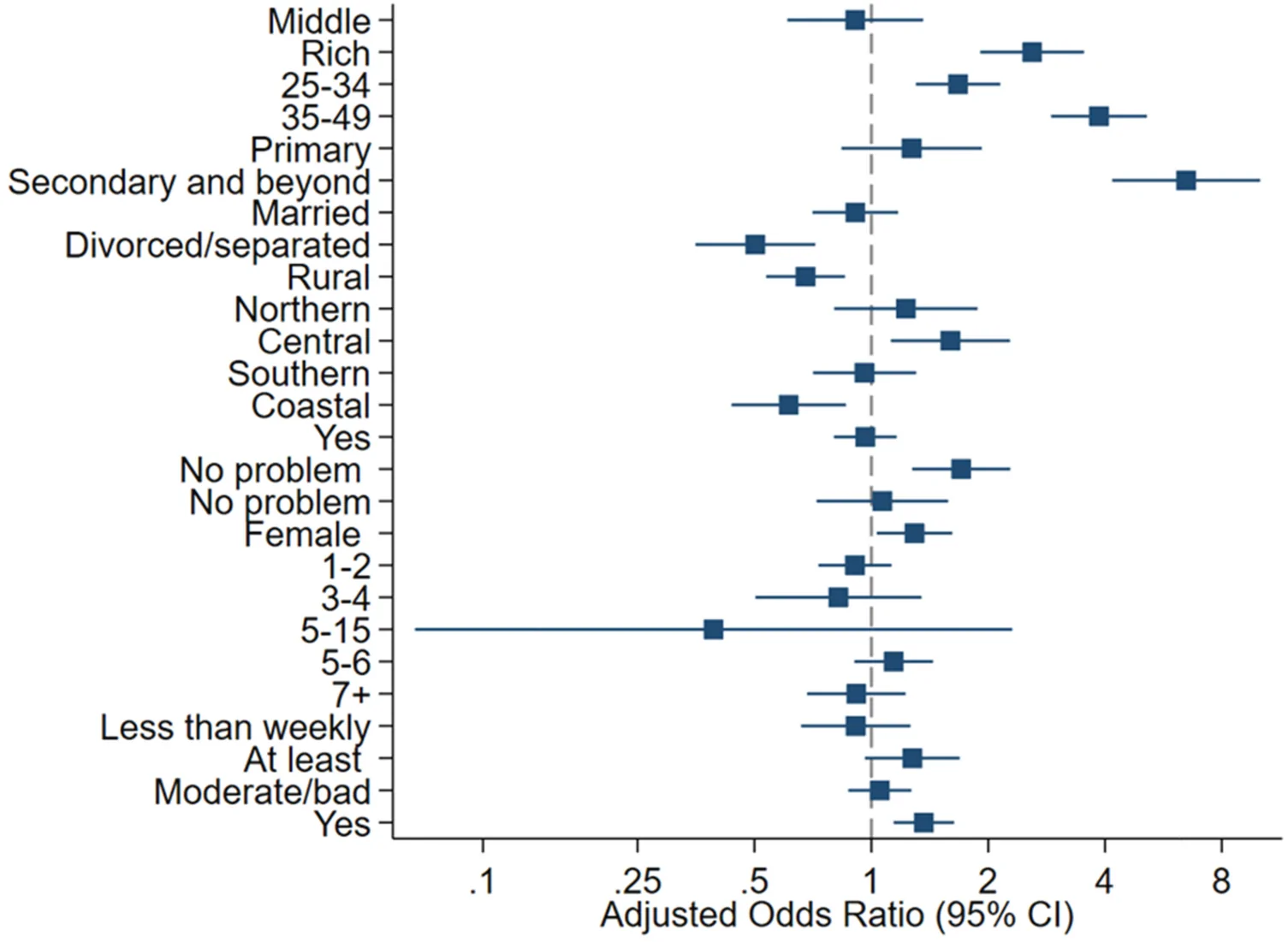
Forest plot of adjusted odds ratios (AOR) with 95% confidence intervals for factors associated with health insurance enrollment among women aged 15–49 years in Tanzania, from the multivariable logistic regression model (TDHS 2022).

To complement the findings presented in Table 4, Figure 1 provides a visual summary of the adjusted odds ratios (AORs) and 95% confidence intervals from the final multivariable logistic regression model, illustrating the magnitude and direction of the associations between the explanatory variables and health insurance enrolment among women aged 15–49 years in Tanzania.

Each horizontal line represents one independent variable from the multivariable logistic regression. For each variable: The square (▪) is the Adjusted Odds Ratio (AOR), the horizontal line is the 95% Confidence Interval (CI) and the vertical dashed line at AOR=1 represents no association.

## Discussion

This study aimed to determine the association between wealth status and health insurance enrollment among women aged 15–49 years in Tanzania. The overall enrollment rate of 5.8% is unacceptably low and consistent with the general struggle in SSA, where only approximately 5% of women of reproductive age in the Democratic Republic of Congo have access to health insurance (18). It is also aligned with the low coverage reported for East Africa, estimated at approximately 7.6% (8), and exceeds the 1.5% reported among women of reproductive age in Malawi (19). This pattern is a manifestation of the inverse care law (20), whereby the availability of good medical care tends to vary inversely with the need for it in the population. It also violates the equity principle of UHC and confirms that health financing efforts are failing to reach the most vulnerable demographic groups. The reliance on OOP payments caused by low insurance coverage serves as a significant financial barrier to accessing healthcare services, and must be addressed as Tanzania implements its new mandatory health insurance policy.

Wealth status, the primary factor of interest in this study showed a positive association with health insurance enrollment: women who belonged to rich households had approximately 2.6 times higher odds of being enrolled in health insurance than those from poor households. This finding highlights a clear wealth-based inequity in access to financial protection against catastrophic health expenditure (5). Wealthier women were significantly more likely to afford insurance premiums, reflecting the positive association between wealth status and enrollment observed elsewhere (18). This is consistent with studies from other settings that found women from rich households to be more likely to own health insurance (8, 18, 19, 21). Increased age, higher educational attainment, visiting a health facility in the past 12 months, living close to a health facility, and coming from a female-headed household were also positively associated with enrollment, while being divorced or separated and residing in rural areas were negatively associated.

Interestingly, the association between wealth and insurance enrollment did not follow a simple linear gradient. Women in the middle wealth category were not significantly different from poor women after adjustment (AOR=0.91, ns), whereas women in the rich category had substantially higher odds of enrollment (AOR=2.59). A similar threshold pattern was observed for education, where primary education was not significantly different from no formal education (AOR=1.27, ns), while secondary or higher education showed a markedly higher odds ratio (AOR=6.47). These findings suggest that a minimum socioeconomic threshold may be necessary before women are able to afford insurance premiums or effectively navigate enrollment procedures, rather than a gradual increase in enrollment across all socioeconomic levels. This has an important policy implication: interventions aimed only at the middle wealth or primary-education groups may have limited impact on enrollment unless they address the specific threshold (for example, premium subsidies) that separates the rich/secondary-educated group from the rest.

Age influenced enrollment, with women in the 35–49 age group having significantly higher odds of enrollment compared with the youngest group (15–24 years). This suggests that older women of reproductive age, possibly due to greater financial stability, increased health risk awareness, or dependency on a spouse's work-related insurance are more likely to be covered (18).

Education was the strongest predictor found in the study: women with secondary or higher education had approximately 6.5 times higher odds of enrollment than those with no formal education. Higher education likely translates to better employment opportunities, higher disposable income for premiums, and improved understanding of the benefits of health insurance. These findings are globally consistent with (8, 18, 21, 22). Existing research has widely identified wealth and education as key factors associated with health service use and insurance uptake (22, 23). Higher education leads to better-paying jobs, enabling individuals to afford premiums, while greater wealth removes the primary financial barrier to access (24).

Women residing in rural areas had significantly lower odds of enrollment compared with those in urban areas. Urban areas generally offer better formal sector employment opportunities (which often include mandatory or subsidised insurance), better access to information, and more established financial systems for premium collection, leading to higher coverage rates (19). This finding is widely supported by comparative studies in Tanzania, East Africa, and other LMICs (8, 18, 19). The difference is largely attributed to the concentration of wealth and higher educational attainment in urban areas. Urban residents are more likely to be in formal employment automatically enrolled in contributory schemes such as the NHIF. In contrast, rural populations rely heavily on the Community Health Fund (CHF), which often struggles with voluntary enrollment, retention, and limited coverage. The perceived or actual poor quality of health services in rural areas also weakens demand for insurance, as it reduces the perceived benefit of paying premiums.

Women residing in the Central zone had significantly higher odds of enrollment compared with those in the Lake zone. This indicates geographical disparities in enrollment, possibly reflecting differences in regional insurance scheme implementation, economic activities, or government and donor programme concentration. This aligns with a study conducted in Tanzania which showed the Central zone to have higher service utilisation linked to health insurance ownership (25). In contrast, the Coastal zone had lower enrollment than the Lake zone. Coastal communities often rely on resource-based livelihoods such as fishing and the informal sector (petty trade, small businesses), which generate low and irregular incomes, making it difficult to afford voluntary annual premiums. Additionally, coastal communities may face unique geographical barriers, including remote and island locations, poor road conditions, and limited transport infrastructure, which reduce access to and perceived benefit of health facilities.

Health service utilisation was a significant predictor of health insurance coverage. Women who had visited a health facility in the past 12 months were significantly more likely to be enrolled. This may reflect that prior interaction with the healthcare system serves as a trigger for enrollment—through facility staff promoting insurance or women learning first-hand about the high costs of care. The strong link between facility visits and insurance ownership suggests that health facilities are a key entry point for expanding coverage in Tanzania. However, it is important to note that this association may be bidirectional, as health insurance has also been reported to facilitate healthcare utilisation (25, 26). While reference (26) uses the same 2022 TDHS dataset, that study examines a distinct outcome healthcare access more broadly, and treats health insurance as one of several explanatory factors, whereas the present study examines health insurance enrollment itself as the outcome, with wealth status as the primary explanatory variable of interest; the two analyses therefore address different research questions using largely non-overlapping model specifications. Similar findings have been reported in other resource-limited settings (8, 27).

Women who perceived distance to a health facility as not a big problem had significantly higher odds of enrollment. This is consistent with well-established determinants of healthcare utilisation and insurance uptake in LMICs (28). Women who live closer to health facilities are more likely to enrol because they perceive a clearer utility in the insurance, knowing they can readily access and use the services offered. Similar findings have been reported elsewhere (27, 28).

Women from female-headed households had significantly higher odds of enrollment. This finding can be understood through the lens of women's empowerment and targeted scheme design. In female-headed households, women are the primary economic and health decision-makers, allowing them to prioritise the health needs of the family. A female household head may also be acutely aware of the risk of catastrophic health expenditure due to limited income sources, making her more proactive in seeking financial protection through insurance. Studies in SSA have shown that women's household decision-making autonomy is positively and significantly associated with higher odds of health insurance enrollment (29). Supporting evidence from Ghana indicates that females are more likely to enroll in health insurance than their counterparts (30), though a contradictory finding from Zambia indicated that being female was associated with lower health insurance coverage (31).

Women who were divorced or separated had lower odds of enrollment than those who were never married. The literature generally shows that all unmarried women have lower odds of coverage compared with married women, as marriage often provides a dual-income and shared-risk household structure (19). The lower odds for the divorced/separated group compared with the never-married group suggest that this status represents a more severe financial shock. Divorce or separation often leads to the loss of spousal financial support, forcing women to become sole income providers while simultaneously shouldering childcare and household expenses, making insurance premiums a low priority.

### Policy implications

The observed findings have several implications for policy. Health facilities should serve as vital entry points for expanding insurance coverage: trained personnel (such as Community Health Officers or registration clerks) should identify uninsured patients at registration and assist with immediate enrollment. Clear, accessible information about schemes such as the iCHF and NHIF should be provided at all facilities. Subsidised or free enrollment should be provided for poor women identified at health facilities, linking them directly to social protection programmes including TASAF. Facility visits can serve as learning moments to build awareness of financial protection through insurance, using patients' own bills to demonstrate how insurance reduces OOP expenses.

Geographical disparities in enrollment require tailored approaches. The implementation of universal health insurance in Tanzania should be planned in a way that accounts for regional differences in economic activity, scheme administration, and access to financial systems. Qualitative research to explore the underlying reasons for non-enrollment among women is also recommended to complement these quantitative findings.

The zone-level differences observed here, particularly the higher enrollment in the Central zone and lower enrollment in the Coastal zone, warrant further investigation. Future studies using these TDHS data could formally test wealth-by-zone and education-by-zone interaction terms, or stratify the multivariable model by zone, to establish whether the effects of wealth and education on enrollment vary by geography; such analyses were beyond the scope of the present study but represent a natural extension of these findings.

### Strengths and limitations

The study used secondary data from the 2022 TDHS, which is nationally representative, ensuring that findings can be generalised to all women of reproductive age (15–49 years) in Tanzania. Analysis was conducted on a weighted sample of over 15,254 women, enhancing statistical power, precision, and reliability of prevalence estimates. By focusing on women aged 15–49 years, the study provides timely insights into health insurance coverage among a group central to national health and development goals.

However, the TDHS data are cross-sectional, meaning that only associations not causal relationships can be established. Health insurance status was self-reported, introducing a risk of recall or social desirability bias. The TDHS wealth index is a composite measure of long-term household assets and may not accurately reflect current disposable income, which is the critical determinant of the ability to pay voluntary health insurance premiums. In addition, the TDHS does not distinguish between formal and informal employment, preventing assessment of differences between contributory and voluntary insurance schemes. Additionally, the survey does not measure respondents' awareness or knowledge of available health insurance schemes, which may influence enrollment decisions. Finally, the quantitative nature of the secondary data analysis is unable to capture underlying qualitative factors influencing enrollment, such as perceived quality of care, distrust in scheme management, or cultural barriers.

## Conclusion

Health insurance enrollment among women of reproductive age in Tanzania is low at 5.8%. There was a significant positive association between wealth status and health insurance enrollment. Belonging to a rich household, being aged 35–49 years, having secondary or higher education, visiting a health facility in the past 12 months, perceiving distance to a health facility as not a problem, and coming from a female-headed household were all positively associated with enrollment. Being divorced or separated and residing in rural areas were negatively associated with enrollment. These findings are particularly relevant as Tanzania implements its new mandatory health insurance policy, and underscore the need for targeted interventions, including social protection linkages and facility-based enrollment strategies to reach the most vulnerable women and advance the goal of universal health coverage. Therefore, the Government of Tanzania should prioritise targeted premium subsidies for poor households, strengthen facility-based enrollment, expand community awareness campaigns, improve access in underserved rural areas, and integrate health insurance enrollment with existing social protection programmes such as TASAF. These interventions will accelerate progress towards universal health coverage (UHC) while reducing socioeconomic inequalities in insurance enrollment.

## Data Availability

Publicly available datasets were analyzed in this study. This data can be found here: DHS programme custodian (https://www.dhsprogram.com/).
